# The Relationship Between Physical Activity and Mobile Phone Addiction Among Adolescents and Young Adults: Systematic Review and Meta-analysis of Observational Studies

**DOI:** 10.2196/41606

**Published:** 2022-12-14

**Authors:** Wen Xiao, Jinlong Wu, Joanne Yip, Qiuqiong Shi, Li Peng, Qiwen Emma Lei, Zhanbing Ren

**Affiliations:** 1 College of Physical Education Shenzhen University Shenzhen China; 2 School of Fashion and Textiles Hong Kong Polytechnic University Hong Kong China; 3 College of Physical Education Southwest University Chongqing China

**Keywords:** mobile phone addiction, physical activity, adolescents, young adults, systematic review, phone addiction, association, correlation, phone use

## Abstract

**Background:**

Previous studies have reported a potential negative correlation between physical activity (PA) and mobile phone addiction (MPA) among adolescents and young adults. To date, the strength of this correlation has not been well characterized.

**Objective:**

This review and meta-analysis aimed to synthesize available empirical studies to examine the correlations between PA and MPA among adolescents and young adults. We also explored several potential moderators, including time of data collection, country or region, and type of population, associated with the relationship between PA and MPA.

**Methods:**

Four electronic databases (PubMed, Scopus, PsycINFO, and Web of Science) were searched from database inception to March 2022 to identify relevant studies. The pooled Pearson correlation coefficients and their corresponding 95% CIs for the relationship between PA and MPA were calculated using the inverse variance method. The methodological quality of the included cross-sectional studies was determined based on the Joanna Briggs Institute appraisal checklist. The study conformed to the PRISMA (Preferred Reporting Items for Systematic Review and Meta-analyses) guidelines.

**Results:**

In total, 892 relevant articles were identified, of which 22 were selected based on the inclusion and exclusion criteria. The final meta-analysis included 17 of the 22 studies. Results of random effects modeling revealed a moderate correlation between PA and MPA among adolescents and young adults (summary *r*=–0.243, *P*<.001). Sensitivity and publication bias analyses further demonstrated the robustness of our results. All the included studies were scored as high quality with a low risk of bias. Subgroup analysis further indicated that none of the hypothesized moderators (time of data collection, country or region, and type of population) significantly affected the relationship between PA and MPA, as confirmed by the mixed effects analysis. In addition, in the data collection subgroups, medium effect sizes were obtained for data collected before COVID-19 (*r*=–0.333, *P*<.001) and data collected during COVID-19 (*r*=–0.207, *P*<.001). In subgroup analyses for country or region, the correlation coefficient for China and other developing regions showed a similarly moderate effect size (*r*=–0.201, *P*<.001 and *r*= –0.217, *P*<.001, respectively). However, the effect sizes for developed regions were not significant (*r*=–0.446, *P*=.39). In a subgroup analysis based on the type of population, we found that the effect size for young adults was moderate (*r*=–0.250, *P*<.001). However, that of adolescents was not significant (*r*=–0.129, *P*=.24).

**Conclusions:**

Our results demonstrate a moderately negative relationship between PA and MPA among young adults. The strength of this relationship was not influenced by the time of data collection, country or region, or type of population.

## Introduction

Mobile phone addiction (MPA) is defined as an addictive behavior in which individuals show uncontrollable use of mobile phones that severely impairs their physical, psychological, and social functions [1,2]. Generally, MPA is considered a negative behavior that is socially inappropriate, or even hazardous, in circumstances such as driving, walking, and unauthorized live streaming [3]. It is also categorized as a behavioral addiction (ie, a nonsubstance addiction) that can potentially cause physical, emotional, and financial harm [4,5].

Previous epidemiological surveys of MPA in different countries and regions in the past 5 years have revealed a high rate of MPA among adolescents and young adults. Recent surveys have also found that the rate of MPA among Brazilian adolescents aged 15 to 18 years was approximately 70.3% [6]. The rate of MPA among college students in Hainan province in China aged 18 to 26 years was 40.5% [7], the rate among Egyptian college students with average age of 18 to 21 years was 64.2% [8], and the rate among college students in a regional city in India with a mean age of 20.1 (SD 1.3) years was 39% to 44% [9].

Numerous investigations have demonstrated that MPA negatively affects mental health by causing anxiety [10] and depression [11,12], affecting sleep quality [13-15] and cognitive function [16], and causing muscle pain [17,18], thereby affecting work productivity and the quality of life of individuals. Thus, MPA is now considered an important worldwide public health topic [19]. MPA has been exacerbated by the spread of SARS-CoV-2 in the recent past and restrictions imposed on social gatherings. This has caused negative psychological effects (eg, anxiety, depression, frustration, fear, and stress) in many individuals [20,21]. As a consequence, the overuse of smartphones, social media, and video gaming has increased as people have found mobile phones to be a coping mechanism to alleviate negative emotions [22,23]. It has been shown that adolescents and young adults are more likely to use mobile phones excessively [24] due to their mental immaturity and lower ability to self-regulate compared to middle-aged and older adults [25,26].

A variety of factors that influence MPA have been explored to develop interventions for preventing MPA in young populations, including physical activity (PA). Data show that PA has broad health benefits, including prolonged life expectancy and better physical and psychological well-being [27]. The World Health Organization recommends that adolescents participate in at least 60 minutes of moderate to vigorous PA daily and 150 to 300 minutes of moderate to vigorous PA per week [28]. A previous meta-analysis showed that PA, including tai chi, basketball, badminton, dance, running, and bicycling, had positive effects on individuals with smartphone addiction [29].

Some cross-sectional studies have predicted that higher levels of PA may reduce rates of MPA among adolescents and young adults, suggesting that there might be a negative correlation between PA and MPA [30-32]. A study in China found a significant negative correlation between PA and MPA in adolescents (ie, people aged 10 to 19 years), which indicates that active participation in PA is a potential strategy to reduce MPA levels [33]. Similar findings were obtained in a study of young adults [30,34]. However, a weak relationship has been reported between PA and MPA among young adults aged 18 to 24 years in other research [35]. Physical inactivity (ie, sedentariness) has been demonstrated to increase the risk of MPA due to the prolonged use of mobile phones. There is evidence that sedentary behaviors and low PA levels are strong predictors of time spent using smartphones [36-38] in adolescents and adults.

To the best of our knowledge, no systematic review and meta-analysis has been conducted to examine the correlation between PA and MPA. Thus, an up-to-date literature review of previous findings on the relationship between PA and MPA is needed. This review identified three knowledge gaps. First, previous findings regarding the strength of the correlation coefficient between PA and MPA in adolescents and young adults are inconsistent. Only one, small-scale systematic review [39] reported a negative correlation between PA and MPA in adolescents. This finding cannot be explained without a quantitative analysis [39]. Second, during the COVID-19 pandemic, isolation policies reduced outdoor PA and increased psychological stress among young adults, which may have increased MPA. However, whether the correlation between PA and MPA was influenced by the pandemic is unclear. Third, as mentioned above, the prevalence of MPA differs across countries and regions. Nevertheless, the question of whether the correlation between PA and MPA is influenced by country or region has remained underexplored.

Therefore, this systematic review and meta-analysis is timely. We sought to examine the overall correlation between PA and MPA and address an important research topic. Furthermore, factors such as the time of data collection (ie, before or during COVID-19), country or region, and type of population (adolescents and young adults) are potential variables influencing the correlation between PA and MPA that we explored and examined with a subgroup analysis.

## Methods

### Protocol Registration

This systematic review and meta-analysis was conducted in line with the PRISMA (Preferred Reporting Items for Systematic Review and Meta-Analyses) guidelines [40].

### Search Strategy

We searched 4 electronic databases (PubMed, Scopus, PsycINFO, and Web of Science) from database inception until March 26, 2022, to identify relevant studies. A manual search was conducted of the retrieved publications to identify potentially missing studies. The search strategy consisted of 2 strings of keywords, including PA- and MPA-related terms. These included the following: (“cell phone” OR “cell phones” OR “cellular phone” OR “cellular phones” OR “cellular telephone” OR “cellular telephones” OR “mobile devices” OR “mobile phone” OR “smart phone” OR “smartphone”) AND (“addiction” OR “dependence” OR “dependency” OR “abuse” OR “addicted to” OR “overuse” OR “problem use” OR “compensatory use”) OR (“problematic smartphone use” OR “problematic smart phone use” OR “problematic mobile phone use” OR “problematic cell phone use” OR “problematic cellular phone use” OR “Nomophobia” OR “Phubbing” OR “fear of missing out” OR “FoMO” OR “smartphone separation anxiety” OR “smartphone use disorder” OR “compulsive mobile phone use”) AND (“physical activity” OR “walk*” OR “exercise*” OR “physical activity*” OR “strength training” OR “resistance training” OR “resistance exercise*” OR “conditioning muscle” OR “training” OR “leisure training” OR “leisure activities” OR “physical fitness” OR “motor activity”). The detailed search strategy is presented in Multimedia Appendix 1. We manually performed a complementary Google search using the abovementioned keyword combinations to broaden the results on September 20, 2022. Secondary searches were performed by manually screening reference lists of included studies and tracking cited articles to ensure no relevant study was omitted.

The identified and retrieved studies were imported into EndNote X7 software (Thompson Reuters). Duplicates were excluded using the deduplication function in Endnote. This screening and processing was conducted by 2 reviewers, who independently read the titles and abstracts and assessed the studies against predetermined inclusion criteria. The full text of the included studies was also independently examined by the 2 reviewers. Inclusion checklists were completed for each study, along with details on the decision to exclude. The reference list of each included study and the articles cited were thoroughly reviewed to ensure that no relevant studies were missed. At all stages, any discrepancies in the results obtained were resolved through consensus or by involving a third reviewer.

### Inclusion Criteria and Study Selection

#### Population

A study was deemed eligible if it included healthy adolescents or young adults aged between 11 and 24 years [41].

#### Exposure and Outcome

Data on PA were collected using measurement tools that included self-reported scales, questionnaires, and accelerometers. Data on different aspects of PA, such as steps taken; time spent each day engaging in light, moderate, and vigorous PA; and PA in different scenarios (ie, for leisure, with family, during active travel, or for work) were recorded. Measurements of MPA levels were collected using internationally used scales or questionnaires (eg, the MPA tendency scale, the mobile phone addiction tendency scale, or the smartphone addiction scale). The contents of the MPA measurement questionnaires or other questionnaires were required to include withdrawal, loss of control and escape, and other MPA symptoms. Studies that only provided data on the duration of mobile phone use were excluded.

#### Study Design

Quantitative observational (cross-sectional and cohort/longitudinal) studies were included.

#### Other Criteria

Studies were included if they were published in peer-reviewed journals and were written in English. If 2 studies were based on the same data set, the study published earlier was selected for inclusion in the review.

### Exclusion Criteria

Case-control studies were excluded because they examined specific groups that were beyond the scope of this review. Furthermore, reviews, meta-analyses, commentaries, replies, clinical guidelines, conference abstracts, theses, and book chapters were also excluded.

### Data Extraction and Synthesis

**Figure 1:**
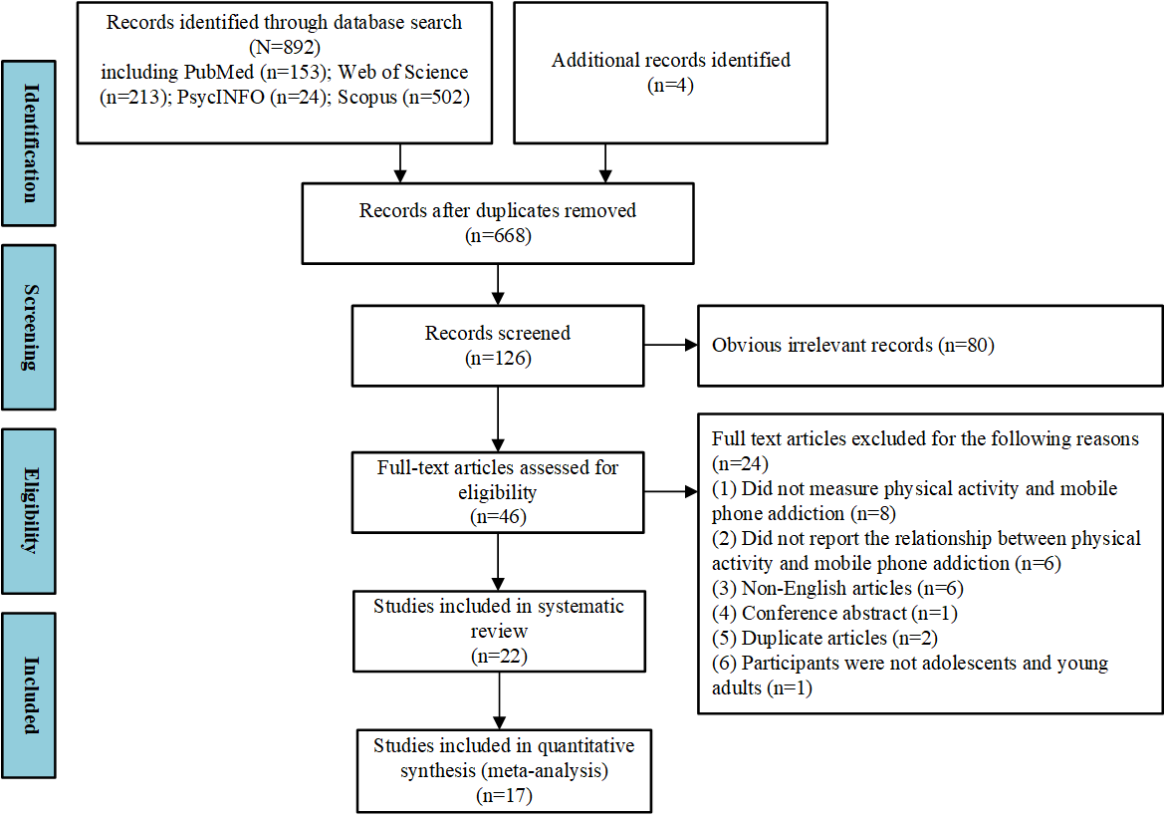
Flow diagram of article screening process.

A total of 892 studies were identified by reading the titles and abstracts. Among these, 46 candidate studies were identified after reading their full text. At this stage, 24 studies were excluded based on the above criteria. The remaining 22 studies were deemed eligible and included in the systematic review. The final meta-analysis included 17 of the 22 studies (Figure 1 shows the details of the article screening process).

Two reviewers independently extracted data from the included articles and entered the data into a form tailored to the requirements of this review. The extracted data included (1) publication details (author, year, and country); (2) sample characteristics (sample size, sex of participants, and type of participant); (3) time of data collection; (4) measurements of PA and MPA; and (5) the main study outcome (ie, correlation coefficient).

### Methodological Quality Assessment

The Joanna Briggs Institute (JBI) appraisal checklist, which has 10 items, was used to examine the methodological quality of the included cross-sectional studies [42]. The studies were given a score of 0 to 2 for each item. Studies with an overall score higher than 70% were considered high quality with a low risk of bias. Details of the scoring criteria applied in the JBI appraisal checklist are presented in Multimedia Appendix 2.

### Data Analysis

All statistical analyses were conducted with Comprehensive Meta-Analysis software (version 3; Biostat Inc).

All data were extracted from the included studies. The pooled Pearson correlation coefficients (with the corresponding 95% CIs) between PA and MPA were calculated with the inverse variance method. Subsequently, the Pearson correlation coefficients were transformed to Fisher *z* scores before the pooled estimate was obtained to calculate variance-stabilized correlation coefficients, as described previously [43] The effect sizes were interpreted in line with recent suggestions concerning correlations for psychometrics with *r*: small (*r*=0.10-0.20), medium (*r*=0.21-0.35), and large (*r*>0.35) [44].

The Cochran Q test and the *I*^2^ statistic were employed to measure heterogeneity across studies. The Cochran Q determines the conformity to the normal distribution of effect sizes. A significant value (*P*<.10) indicates heterogeneity. *I*^2^ is an estimate of the ratio of true heterogeneity in the observed variation. *I*^2^>50% reflects statistically significant between-study heterogeneity [45]. For such studies, the random effects model was used to calculate the summary of the Pearson correlation coefficients with a *P* value <.05 or *I*^2^>50% [44]. Otherwise, the fixed effects model was used [44].

To determine potential moderators of heterogeneity, subgroup analyses were carried out for country or region, population (college students and adolescents), and time of data collection (before or during COVID-19). All subgroup analyses were conducted with a mixed effects analysis. The random effects model was used to summarize the studies within the respective subgroups, and the fixed effects model was used to test for significant differences between the subgroups [46]. Full details of coding forms for the subgroups are provided in Multimedia Appendix 3.

To determine the influence of individual studies on the summary correlation coefficients and test the robustness of the correlations between PA and MPA, sensitivity analyses were conducted by sequentially omitting one study at time [11].

Funnel plots were established to determine the existence of potential publication bias. Additionally, the Begg rank correlation test and Egger linear regression test were performed to determine publication bias, with *P*<.05 indicating significant publication bias [47,48]. In the case of publication bias, the trim-and-fill method was used to adjust for funnel plot asymmetry [11].

Other statistical analyses performed included valid measures of the association between PA and MPA, measured with the correlation coefficient (*r*), standardized regression coefficient (b), unstandardized regression coefficient (β), odds ratio (OR), mean, and SD. To include as many eligible studies as possible, several data transformation steps were used. For studies that reported the mean and SD, the Cohen *d* effect size was calculated and converted to a correlation [49]. Studies that reported relevant ORs with 95% CIs were converted to Cohen *d* effect estimates and then to correlations [50]. The authors of the eligible studies were contacted if potentially relevant data were missing.

## Results

### Descriptive Characteristics

**Figure 2:**
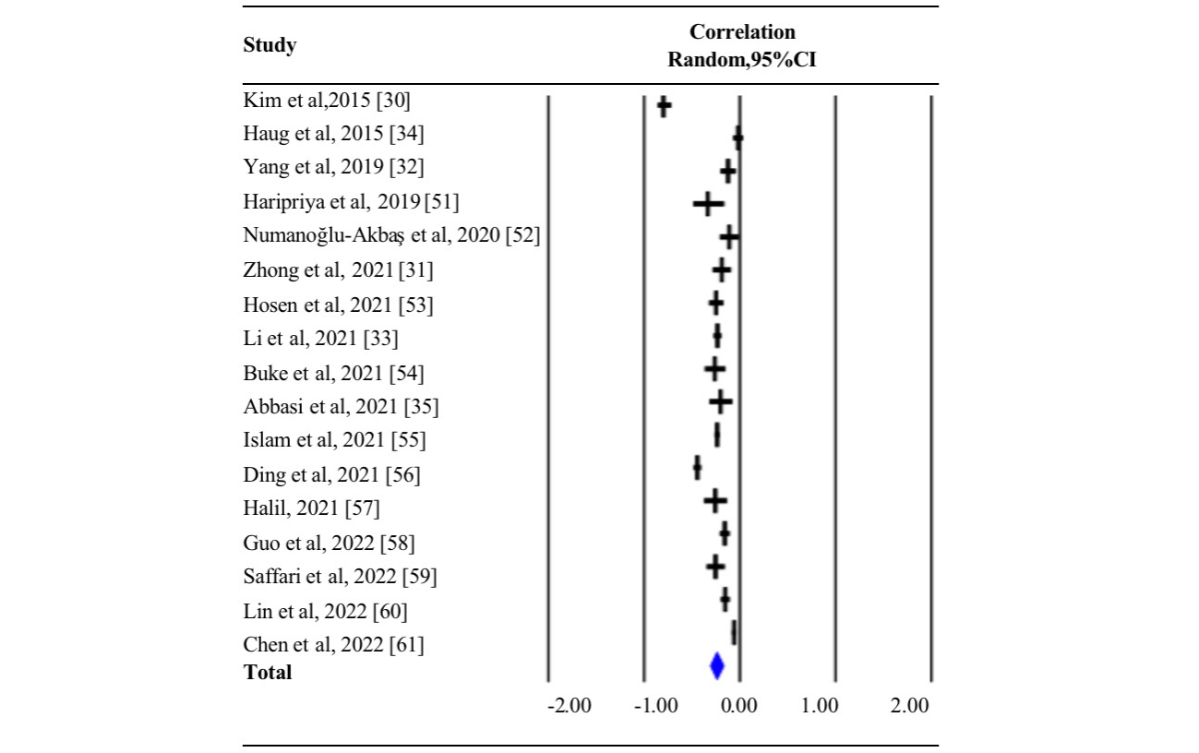
Summary of pooled correlation between physical activity and mobile phone addiction. The blue diamond represents the overall pooled correlation for the random effects model [30-35,51-61].

Table 1 presents a summary of the characteristics of the included studies. Overall, 23,365 participants aged between 15 and 26 years were included. Eighteen studies (numbers 1 to 22) were included in the systematic review, and 17 studies (numbers 1 to 17) were included in the meta-analysis. Moreover, 17 studies reported a correlation between PA and MPA. Considering the high heterogeneity among studies (Q=468.050, *P*<.001; *I*^2^=96.582), the random effects model was used to estimate the effect size of summary *r* (*r*=–0.243; 95% CI –0.309 to –0.175; *P*<.001; Table 2 and Figure 2). This result showed that PA was moderately negatively correlated with MPA.

**Table 1:** Characteristics of the studies included in the review.

Study	Country	Size, n	Male, n	Population	Age (years)	Time period	MPA^a^ measurement	PA^b^ measurement	*r*
1. Kim et al, 2015 [30]	South Korea	110	67	College students	Mean 21.03 (SD 1.61)	2015	SAPS^c^	3D sensor pedometer	–0.798
2. Haug et al, 2015 [34]	Switzerland	1519	732	Adolescents	Range 16-21	Feb 2015 to Jun 2015	SAS-SV^d^	“Outside school: How many hours a week do you exercise or participate in sports that make you sweat or become out of breath?”	–0.019
3. Yang et al, 2019 [32]	China	608	158	College students	—^e^	Dec 2018 to Jan 2019	MPATS^f^	PARS-3^g^	–0.124
4. Haripriya et al, 2019 [51]	India	113	63	College students	Mean 22.15 (SD 1.69)	Apr 2019 to May 2019	SAPS	IPAQ-SF^h^	–0.335
5. Numanoğlu- Akbaş et al, 2020 [52]	Turkey	388	129	College students	Range 17-25	Jan 2019 to Jun 2019	SAS^i^	IPAQ-SF	–0.112
6. Zhong et al, 2021 [31]	China	394	115	College students	—	Jul 29, 2020	CSMDQ^j^	PARS-3	–0.190
7. Hosen et al, 2021 [53]	Bangladesh	601	344	College students	—	Oct 2020 to Nov 2020	SABAS^k^	Physical exercise questions (eg, at least 30 minutes daily walking, cycling, swimming, or other activities regularly)	–0.249
8. Li et al, 2021 [33]	China	2407	280	Adolescents	Mean 16.27 (SD 1.02)	Dec 2020 to Feb 2021	Self-rating questionnaire for adolescent problematic mobile phone use	PA questionnaire A^l^	–0.235
9. Buke et al, 2021 [54]	Turkey	300	166	College students	Mean 21.36 (SD 2.33)	Apr 2020	SAS-SV	IPAQ^m^	–0.262
10. Abbasi et al, 2021 [35]	Malaysia	250	145	College students	—	May 2020	SAS-SV	Physical activity questionnaire B^n^	–0.201
11. Islam et al, 2021 [55]	Bangladesh	5511	3254	College students	Mean 21.20 (SD 1.70)	Jul 2020	SABAS	Questions were asked regarding the engagement in infrequent activities (including home quarantine regular/frequent activities (ie, academic/other studies, social-media use, watching television, household chores, and professional activities)	–0.238
12. Ding et al, 2021 [56]	China	1724	740	College students	Mean 19.56 (SD 0.95)	Sep 2020	MPATS	PARS-3	–0.445
13. Halil, 2021 [57]	Pakistan	236	123	College students	—	2020 to 2021	SAS-SV	IPAQ-SF	–0.258
14. Guo et al, 2022 [58]	China	1433	704	College students	Mean 19.67 (SD 1.62)	Dec 2020 to Feb 2021	MPATS	PARS-3	–0.158
15. Saffari et al, 2022 [59]	Taiwan	391	0	College students	Mean 22.85	Aug 2021 to Sep 2021	SABAS	IPAQ-SF	–0.255
16. Lin et al, 2022 [60]	China	1787	628	College students	Range 18-22	Aug 2020 to Sep 2021	SAS	IPAQ-SF	–0.153
17. Chen et al, 2022 [61]	China	9406	3516	College students	Mean 19.58 (SD 1.07)	Mar 2022 to Apr 2022	MPAS^o^	IPAQ-L^p^	–0.060
18. Venkatesh et al, 2019 [62]	Saudi Arabia	205	101	College students	Mean 23.28	Jan 2016 to Mar 2016	SAS-SV	“Outside school: How many hours a week do you exercise or participate in sports that make you sweat or become out of breath?”	—
19. Xie et al, 2019 [63]	China	2134	917	College students	Mean 19.25 (SD 1.42)	Jun 2014 to Dec 2014	Self-rating questionnaire for adolescent problematic mobile phone use	During the past 7 days, on how many days were you physically active for a total of at least 60 minutes per day?	—
20. Pereira et al, 2020 [64]	Brazil	667	308	Adolescents	Range 13-18	—	SAS-SV	IPAQ-SF	—
21. Tao et al, 2020 [65]	China	4624	2057	College students	Mean 19.91 (SD 1.27)	May 2018 to Jun 2018	Self-rating questionnaire for adolescent problematic mobile phone use	IPAQ-SF	—
22. Zou et al, 2021 [66]	China	251	52	College students	Mean 19.01 (SD 0.85)	Apr 2019 to Jun 2019	Self-rating questionnaire for adolescent problematic mobile phone use	IPAQ-C^q^	—

^a^MPA: mobile phone addiction.

^b^PA: physical activity.

^c^SAPS: Smartphone Addiction Proneness Scale.

^d^SAS-SV: Smartphone Addiction Scale–Short Version.

^e^Not available.

^f^MPATS: Mobile Phone Addiction Tendency Scale.

^g^PARS-3: Physical Activity Rating Scale–3.

^h^IPAQ-SF: International Physical Activity Questionnaire–Short Form.

^i^SAS: Smartphone Addiction Scale.

^j^CSMDQ: College Students Mobile Phone Dependence Questionnaire.

^k^SABAS: Smartphone Application-Based Addiction Scale.

^l^Physical activity questionnaire A was derived from [67].

^m^IPAQ: International Physical Activity Questionnaires.

^n^Physical activity questionnaire B was derived from [68].

^o^MPAS: Mobile Phone Addiction Scale.

^p^IPAQ-L: International Physical Activity Questionnaire–Long Form.

^q^IPAQ C: International Physical Activity Questionnaire–Chinese.

**Table 2:** Statistics for each study^a^.

Study	*r* (total *r*=0.243)	95% CI (total 95% CI –0.309 to –0.175)	*z* (total *z*=–6.810)	*P* value (total *P*<.001)	Weight (total 100%)
Kim et al, 2015 [30]	–0.798	–0.190 to 0.184	–0.031	.98	4.48%
Haug et al, 2015 [34]	–0.019	–0.069 to 0.031	–0.740	.46	6.35%
Yang et al, 2019 [32]	–0.124	–0.202 to –0.045	–3.066	.002	6.06%
Haripriya et al, 2019 [51]	–0.335	–0.486 to –0.165	–3.753	<.001	4.60%
Numanoğlu-Akbaş et al, 2020 [52]	–0.112	–0.209 to –0.013	–2.207	.03	5.81%
Zhong et al, 2021 [31]	–0.190	–0.283 to –0.093	–3.803	<.001	5.82%
Hosen et al, 2021 [53]	–0.249	–0.323 to –0.172	–6.220	<.001	6.05%
Li et al, 2021 [33]	–0.235	–0.272 to –0.197	–11.742	<.001	6.42%
Buke et al, 2021 [54]	–0.262	–0.364 to –0.153	–4.623	<.001	5.62%
Abbasi et al, 2021 [35]	–0.201	–0.317 to –0.079	–3.203	<.001	5.46%
Islam et al, 2021 [55]	–0.238	–0.263 to –0.213	–18.009	<.001	6.50%
Ding et al, 2021 [56]	–0.445	–0.482 to –0.406	–19.848	<.001	6.37%
Halil, 2021 [57]	–0.258	–0.373 to –0.135	–4.029	<.001	5.41%
Guo et al, 2022 [58]	–0.158	–0.208 to –0.107	–6.025	<.001	6.34%
Saffari et al, 2022 [59]	–0.255	–0.345 to –0.160	–5.136	<.001	5.81%
Lin et al, 2022 [60]	–0.153	–0.198 to –0.107	–6.513	<.001	6.38%
Chen et al, 2022 [61]	–0.060	–0.080 to –0.040	–5.825	<.001	6.52%

^a^Heterogeneity: Q=468.050; *P*<.001; *I*²=96.582.

### Subgroup Analysis

As shown in Table 3, the summary correlation coefficient between PA and MPA did not change when stratified by time of data collection, country or region, or type of population (all *P*^b^>.05). However, to allow comparison with other studies, we present model-implied effect sizes for each level of the moderator.

The time of data collection did not significantly moderate the effect sizes (between-subgroup *P*^b^=.14). Notably, the summary correlation coefficient of the studies reporting on data collected before COVID-19 was slightly higher compared with that for data collected during COVID-19. Specifically, the effect sizes for data collection before COVID-19 were moderate, with a 95% CI that did not overlap with 0 (*r*=–0.333, 95% CI –0.466 to –0.187; k=4; *P*^a^<.001), whereas the effect sizes for data collected during COVID-19 were also moderate, with 95% CIs that overlapped with 0 (*r*=–0.207, 95% CI –0.285 to –0.126, k=13; *P*^a^<.001).

Similarly, we did not find significant moderator effect sizes for country or region (between-subgroup *P*^b^=.71). The summary correlation coefficient for both China and other developing regions showed a similarly moderate effect size (China: *r*=–0.201, 95% CI –0.311 to –0.127; k=7; *P*^a^<.001; other developing regions: *r*= –0.217, 95% CI –0.326 to –0.103; k=8; *P*^a^<.001). However, the effect sizes for developed regions with a 95% CI that overlapped with 0 (*r*=–0.446, 95% CI –0.616 to 236, k=2; *P*^a^=.24) were not significant.

In addition, there were no significant moderator effect sizes for the type of population (between-subgroup: *P*^b^=.26). Specifically, we found that the effect sizes for young adults were moderate, with a 95% CI that did not overlap with 0 (*r*=0.250, 95% CI –0.325 to –0.173, k=15; *P*^a^<.001). However, the effect sizes for adolescents were not significant, with a 95% CI that overlapped with 0 (*r*=–0.129, 95% CI –0.333 to 0.086, k=2, *P*^a^=.24).

**Table 3:** Subgroup analyses of summary correlation between PA and MPA. *P*^a^ values for the within-subgroup effect sizes were calculated with the *z* test; *P*^b^ values for between-subgroup differences were calculated with the Q test; and *P*^c^ values for heterogeneity within subgroups were calculated with the Q test.

Moderator		Studies, n	Summary *r* (95% CI)	*P*^a^ value	Heterogeneity		*P*^b^ value
					*I*^2^ (%)	*P*^c^ value	
**Time of data collection**							.14
	Before COVID-19	4	–0.333 (–0.466 to –0.187)	<.001	97.555	<.001	
	During COVID-19	13	–0.207 (–0.285 to –0.126)	<.001	96.438	<.001	
**Country or region**							.71
	Developed regions	2	–0.446 (–0.616 to 0.236)	.39	99.133	<.001	
	China	7	–0.201 (–0.311 to –0.127)	<.001	97.873	<.001	
	Other developing regions	8	–0.217 (–0.326 to –0.103)	<.001	63.310	<.001	
**Population**							.26
	Young adults	15	–0.250 (–0.325 to –0.173)	<.001	96.681	<.001	
	Adolescents	2	–0.129 (–0.333 to 0.086)	.24	97.787	<.001	

### Sensitivity Analyses and Publication Bias

In the analysis that removed studies one at a time, no evident outliers were identified. Thus, the correlation coefficient for removing each study was in the range of *r*=–0.195 to –0.248. This shows that no one study significantly skewed or changed the correlation coefficient or influenced the overall results of the meta-analysis. Therefore, the results were reliable.

**Figure 3:**
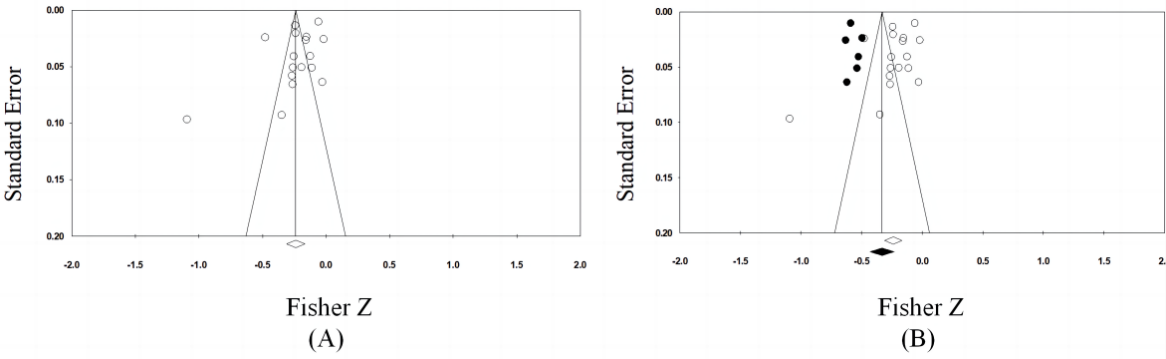
Funnel plots of (A) publication bias and (B) publication bias with trim and fill.

Subjectively speaking, we could not determine the existence of publication bias from the funnel plots for the summary correlation coefficients, as shown in Figure 3A. Studies with a small sample size are unlikely to result in symmetrical distributions of scattered points. The Begg rank correlation tests and Egger linear regression tests showed no significant publication bias (*P*=.62 and *P*=.14, respectively). After the trim-and-fill analysis, the correlation between PA and MPA remained statistically significant (number to trim and fill 6, summary *r*=–0.319, 95% CI –0.40552 to –0.22771; Figure 3B). Therefore, the overall modeling results after correction remained unchanged. Thus, we concluded that there was no publication bias.

### Methodological Quality Assessment

The methodological quality assessment results are shown in Table 4. Notably, the mean scores of the included studies and the other studies were 15.29 (SD 1.53) and 15.40 (SD 0.89), respectively. All included studies were of high quality with a low risk of bias.

**Table 4:** Methodological quality of the studies.

Number	Study	Joanna Briggs Institute appraisal checklist items											Total score (%)		Overall risk of bias
		1	2	3	4	5	6	7	8	9	10				
1	Kim et al, 2015 [30]	1	1	1	1	2	2	2	2	2	1	15 (75)		Low	
2	Haug et al, 2015 [34]	2	0	1	1	2	2	2	2	2	2	16 (80)		Low	
3	Yang et al, 2019 [32]	1	2	1	2	2	2	2	2	2	2	18 (80)		Low	
4	Haripriya et al, 2019 [51]	2	0	1	1	2	2	2	2	2	1	15 (75)		Low	
5	Numanoğlu-Akbaş et al, 2020 [52]	2	0	0	0	2	2	2	2	1	2	13 (65)		Mid	
6	Zhong et al, 2021 [31]	2	2	1	1	2	2	0	2	2	2	16 (80)		Low	
7	Hosen et al, 2021 [53]	1	1	1	1	1	2	2	2	1	2	14 (70)		Low	
8	Li et al, 2021 [33]	2	1	1	1	2	2	2	2	2	2	17 (85)		Low	
9	Buke et al, 2021 [54]	1	1	1	1	2	2	2	2	2	1	15 (75)		Low	
10	Abbasi et al, 2021 [35]	2	1	1	1	2	2	0	1	1	1	15 (75)		Low	
11	Islam et al, 2021 [55]	1	0	1	2	2	2	2	2	1	2	15 (75)		Low	
12	Ding et al, 2021 [56]	2	1	1	1	2	2	0	2	2	1	14 (70)		Low	
13	Halil, 2021 [57]	2	0	1	1	2	2	0	2	2	2	14 (70)		Low	
14	Guo et al, 2022 [58]	2	2	1	1	2	2	2	2	2	2	18 (80)		Low	
15	Saffari et al, 2022 [59]	2	0	1	2	2	2	2	2	2	2	17 (85)		Low	
16	Lin et al, 2022 [60]	2	0	0	0	2	2	2	2	2	1	13 (65)		Mid	
17	Chen et al, 2022 [61]	2	2	0	1	2	2	0	2	2	2	15 (75)		Low	
18	Venkatesh et al, 2019 [62]	1	0	1	1	2	2	2	2	2	1	14 (70)		Low	
19	Xie et al, 2019 [63]	1	0	1	2	2	2	2	2	2	2	16 (80)		Low	
20	Pereira et al, 2020 [64]	1	0	1	2	2	2	2	2	2	2	16 (80)		Low	
21	Tao et al, 2020 [65]	2	1	1	2	2	2	0	2	2	1	15 (75)		Low	
22	Zou et al, 2021 [66]	2	0	1	2	2	2	2	2	2	1	16 (80)		Low	

## Discussion

### Meta-analytic Findings

To the best of our knowledge, this is the first meta-analysis to explore pooled correlation coefficients of PA and MPA. Our analysis of 17 studies found a moderately negative correlation between PA and MPA, with a summary Pearson correlation coefficient of *r=*–0.243. This is in line with a previous review [40]. Sensitivity analyses did not find significant publication bias, indicating that the pooled analyses of the correlation coefficients provided reliable and convincing results. In addition, all the included studies were high quality with a low risk of bias. Subgroup analysis showed that none of the hypothesized moderators (data collection, country or region, and type of population) significantly influenced the relationship between PA and MPA, as confirmed with a mixed effects analysis.

The target subjects of research into MPA are adolescents and young adults, who are relatively less self-disciplined in controlling their frequency of mobile phone use and are more susceptible to smartphone use addiction compared to middle-aged or older adults [29]. Lack of self-control is an essential factor influencing MPA among adolescents and young adults. Previous studies found that self-control regulates the correlation between PA and MPA. Factors such as negative emotions (eg, anxiety [69,70] and loneliness [71,72]) and mental toughness [73,74] have been shown to affect the relationship between PA and MPA. We speculate that these factors may modulate the relationship between PA and MPA.

Results of magnetic resonance imaging studies suggest that MPA is associated with structural brain abnormalities, like other types of addiction dependence. For example, the insula cortex participates in the formation of addictive behaviors, because these behaviors may influence the decision-making process in terms of choosing immediate rewards that are always associated with physiological state while eliciting strong interoceptive signals [75]. Two recent studies reported changes in gray matter volume in this region (ie, the insula cortex) among people with MPA [66,76]. Exercise has been shown to improve brain health [77,78]. Therefore, we hypothesize that the relationship between PA and MPA might be influenced by the structure and function of the insula and even other brain regions.

### Differences in Subgroup Analysis

Notably, the time of data collection did not significantly influence the relationship between PA and MPA. Moreover, a moderate negative relationship was found between PA and MPA among adolescents and young adults before and during the COVID-19 pandemic. According to the compensatory internet use theory, when people encounter psychosocial problems in the real world, they are likely to use the internet or smartphones as a coping mechanism to alleviate negative emotions [79]. The restrictions imposed on participation in social activities and gatherings during the COVID-19 pandemic increased anxiety, depression, and stress levels in people [80]. Therefore, they were more likely to overly rely on their mobile phones to cope with stress. Moreover, recent studies have shown that adolescents and young adults have had low PA levels during the COVID-19 pandemic [81,82]. The target subjects in our study were adolescents and young adults, who are more inclined to use social media for physical training. A previous study found that young-adult Spanish university students used social media apps to improve their high-intensity interval training [82], mind-body activities, and strength exercises. In other words, adolescents and young adults used social media to facilitate their participation in PA during the pandemic [82]. This reduced the time spent sitting for long periods of time and reduced leisure-time screen activities, subsequently reducing the risk of MPA in young people [30].

The present findings demonstrate that country or region do not have a significant moderating role on the relationship between PA and MPA. Notably, a medium-strength negative relationship between PA and MPA has been reported in China and other developing counties among adolescents and young adults. However, this correlation was not found in developed countries. This finding should be interpreted with caution, because it is based on 2 studies from developed countries. These 2 studies were carefully reviewed elsewhere [30,34]. In addition, we found that one of these studies reported a weak negative correlation between PA and MPA [34], whereas the other found a significant, strong negative correlation [30]. It should be noted that the 2 studies were published around the same time. The difference in the correlation results may be due to the type of PA measurement tools used. For instance, one of the studies used a pedometer sensor to measure the level of PA, which is more precise [30]. The influence of measurement errors associated with self-reported PA questionnaires also needs to be acknowledged. The majority of the reviewed studies used self-reported scales or questionnaires; thus, we suggest that accelerometry should be adopted in future studies to obtain more reliable data.

Further analysis revealed that population type did not significantly affect the relationship between PA and MPA. This may be explained by the widespread use of mobile phones. This is especially true for young people, as their ownership rate for smartphones is very high. Additionally, subgroup analysis revealed that there was no significant correlation between PA and MPA among adolescents (*P*=.26). We presume that this might be influenced by the degree of external restrictions on the use of mobile phones. Compared with adults (eg, college students), adolescents are subjected to more control and restriction measures on mobile phone use by their parents, schools, and even by the mobile phone apps themselves. Therefore, they are less likely to influence the correlation [83]. These findings, however, should be interpreted with caution, because only 2 studies on adolescents were analyzed.

### Limitations and Strengths

In conclusion, our study indicates that a low PA level contributes to MPA behavior. This is because low PA encourages a sedentary lifestyle among young adults. The PA guidelines of the World Health Organization encourage individuals of different ages to participate in PA. Previous studies have shown that increasing the PA level of young adults can reduce MPA behavior. We recommend higher PA levels than those stipulated in the guidelines of the World Health Organization, because more PA could bring more mental health benefits. From a practical perspective, the findings of this study may help to inform countermeasures to prevent MPA behavior among adolescents and young adults amid the COVID-19 pandemic and future public health crises.

All previous findings were objectively stated, analyzed, and interpreted using an appropriate research design. All original data were retained to provide a reference for future research. The repeatability and reproducibility of our analyses have been ensured. However, there are some limitations and potential sources of bias that need to be noted. First, only studies published in English were included in our meta-analysis. Second, the studies mainly provided cross-sectional data, which do not allow determination of causality in the relationship between PA and MPA. Third, we only analyzed a young population. Fourth, no study reported moderating variables between PA and MPA. Finally, although a sensitivity analysis was conducted, sources of bias were identified, and our results should thus be interpreted with caution. Further case-control and cohort studies are needed to test the benefits of PA on MPA in young adults.

### Conclusion

Our findings demonstrate a moderate negative relationship between PA and MPA among young adults. The strength of the relationship between PA and MPA did not differ by time of data collection, country or region, or type of population.

## Appendix

Multimedia Appendix 1 Detailed search strategy.

Multimedia Appendix 2 Details of the scoring criteria in the JBI appraisal checklist. JBI: Joanna Briggs Institute.

Multimedia Appendix 3 Full details of coding forms for the subgroups.

## Abbreviations

JBI: Joanna Briggs Institute
MPA: mobile phone addiction
OR: odds ratio
PA: physical activity
PRISMA: Preferred Reporting Items for Systematic Review and Meta-analyses
