# Supplementary material for: The Relationship Between Physical Activity and Mobile Phone Addiction Among Adolescents and Young Adults: Systematic Review and Meta-analysis of Observational Studies
Source: JMIR Public Health Surveill. 2022 Dec 14;8(12):e41606. doi: 10.2196/41606 (PMC9798266; doi:10.2196/41606)
Supplement: Multimedia Appendix 1 [file publichealth_v8i12e41606_app1.docx]

**Appendix Table 1.** Detailed search strategy

| Database | Search strategy | Limits |
| --- | --- | --- |
| PsyclNFO: 24 (By using abstract)  Pubmed: 153 (By using title/abstract) Scopus: 502 (By using title/abstract/key)  Web of science: 213 (By using topic) | (“cell phone”OR“cell phones”OR“cellular phone” OR“cellular phones” OR“cellular telephone”OR“cellular telephones”OR“mobile devices”OR“mobile phone”OR“smart phone” OR“smartphone”) AND (addiction OR dependence OR dependency OR abuse OR“addicted to”OR overuse OR“problem use”OR“compensatory use”) OR (“problematic smartphone use”OR “problematic smart phone use”OR“problematic mobile phone use”OR“problematic cell phone use”OR“problematic cellular phone use”OR Nomophobia OR Phubbing OR“fear of missing out”OR FoMO OR“smartphone separation anxiety”OR“smartphone use disorder”OR“compulsive mobile phone use”)AND (“physical activity”OR walk* OR exercise* OR“physical activit*”OR“strength training”OR“resistance training”OR“resistance exercise*”OR“conditioning muscle”OR training OR“leisure training”OR“leisure activities”OR “physical fitness”OR“motor activity) | -Human;  -English language |
|  |  |  |
|  |  |  |
