# Supplementary material for: The Relationship Between Physical Activity and Mobile Phone Addiction Among Adolescents and Young Adults: Systematic Review and Meta-analysis of Observational Studies
Source: JMIR Public Health Surveill. 2022 Dec 14;8(12):e41606. doi: 10.2196/41606 (PMC9798266; doi:10.2196/41606)
Supplement: Multimedia Appendix 2 [file publichealth_v8i12e41606_app2.docx]

**Appendix Table 1.** Details of the scoring criteria in the JBI appraisal checklist

| Number | Entry | Evaluation standard |
| --- | --- | --- |
| 1 | Is the purpose of the study clear? Is the foundation of the thesis sufficient? | **0**:Out of order；  **1**:Mentioned, but not described in detail；  **2**: Detailed, comprehensive and correct description。 |
| 2 | How was the study population selected (Were the study subjects randomly selected, was stratified sampling used to increase sample representation)？ |  |
| 3 | Are inclusion and exclusion criteria clearly described? |  |
| 4 | Does it clearly characterize the sample? |  |
| 5 | Are the data collection tools reliable and valid? (If investigators are used, how about the repeatability of the survey results)？ |  |
| 6 | What are the measures to verify the authenticity of the information? |  |
| 7 | Are ethical issues considered? |  |
| 8 | Is the statistical method correct? |  |
| 9 | Are the statements of the findings appropriate and accurate (are the results and inferences distinguished, and are the results faithful to the data rather than inferences)? |  |
| 10 | Is the importance of the research clearly stated? |  |
