# Supplementary material for: The Relationship Between Physical Activity and Mobile Phone Addiction Among Adolescents and Young Adults: Systematic Review and Meta-analysis of Observational Studies
Source: JMIR Public Health Surveill. 2022 Dec 14;8(12):e41606. doi: 10.2196/41606 (PMC9798266; doi:10.2196/41606)
Supplement: Multimedia Appendix 3 [file publichealth_v8i12e41606_app3.docx]

**Appendix Table 3.** Full details of coding forms for the subgroups

| **Reference** | **Time of data collection** | **Country or region** | **Population** |
| --- | --- | --- | --- |
| Kime et al., 2015 | 2015→Before the COVID-19 | South Korea→ [developed country](http://www.baidu.com/link?url=8Dw0j1zfLQsivfzz3AVQ_pXJHkAaoMsAbaJg-d2yHBH_1-2jepTdMF-QypuouzRSJMw6-_eHbfvkMzwJrZXXPAMEo6H0CNDcPbwz8qqvNuf4Mv3V4eBctO5ECZ_pOt9B) | College students（21.03± 1.61 age）→ Young Adults |
| Haug et al., 2015 | 2015.2-2015.6→Before the COVID-19 | Switzerland→[developed country](http://www.baidu.com/link?url=8Dw0j1zfLQsivfzz3AVQ_pXJHkAaoMsAbaJg-d2yHBH_1-2jepTdMF-QypuouzRSJMw6-_eHbfvkMzwJrZXXPAMEo6H0CNDcPbwz8qqvNuf4Mv3V4eBctO5ECZ_pOt9B) | Adolescents（16 - 21 age）→among Adolescents |
| Yang et al., 2019 | 2018.12-2019.1 →  Before the COVID-19 | China→ [developing country](https://www.baidu.com/link?url=gpxre6LNEajmcBWVAxBRE_lGvyQEb1T3VICjVuhq7pJ3M_AOsX_3zEhqDwAllNkjmRh5s2EyiUhipyeH7FTCC39izPWoteQDaiQik5ZWfB8yhCrdqba_wzooZzmK7EoJ&wd=&eqid=dfb8ae4300001d140000000663314fb4) | College students→ Young Adults |
| Haripriya et al., 2019 | 2019.4-2019.5→Before the COVID-19 | China→ [developing country](https://www.baidu.com/link?url=gpxre6LNEajmcBWVAxBRE_lGvyQEb1T3VICjVuhq7pJ3M_AOsX_3zEhqDwAllNkjmRh5s2EyiUhipyeH7FTCC39izPWoteQDaiQik5ZWfB8yhCrdqba_wzooZzmK7EoJ&wd=&eqid=dfb8ae4300001d140000000663314fb4) | College students(22.15±1.69  age)→Young Adults |
| Numanolu-Akba., 2020 | 2019.1-2019.6→Before the COVID-19 | Bangladesh→[developing country](https://www.baidu.com/link?url=gpxre6LNEajmcBWVAxBRE_lGvyQEb1T3VICjVuhq7pJ3M_AOsX_3zEhqDwAllNkjmRh5s2EyiUhipyeH7FTCC39izPWoteQDaiQik5ZWfB8yhCrdqba_wzooZzmK7EoJ&wd=&eqid=dfb8ae4300001d140000000663314fb4) | College students(17-25 age)→Young Adults |
| Zhong et al., 2021 | 2020.7.29→During the COVID-19 | China→ [developing country](https://www.baidu.com/link?url=gpxre6LNEajmcBWVAxBRE_lGvyQEb1T3VICjVuhq7pJ3M_AOsX_3zEhqDwAllNkjmRh5s2EyiUhipyeH7FTCC39izPWoteQDaiQik5ZWfB8yhCrdqba_wzooZzmK7EoJ&wd=&eqid=dfb8ae4300001d140000000663314fb4) | Adolescents→among Adolescents |
| Kheradm et al. 2021 | 2020.10-2020.11→During the COVID-19 | Turkey→[developing country](https://www.baidu.com/link?url=gpxre6LNEajmcBWVAxBRE_lGvyQEb1T3VICjVuhq7pJ3M_AOsX_3zEhqDwAllNkjmRh5s2EyiUhipyeH7FTCC39izPWoteQDaiQik5ZWfB8yhCrdqba_wzooZzmK7EoJ&wd=&eqid=dfb8ae4300001d140000000663314fb4) | College students→Young Adults |
| Li et al., 2021 | 2020.12-2021.2→During the COVID-19 | Malaysia→[developing country](https://www.baidu.com/link?url=gpxre6LNEajmcBWVAxBRE_lGvyQEb1T3VICjVuhq7pJ3M_AOsX_3zEhqDwAllNkjmRh5s2EyiUhipyeH7FTCC39izPWoteQDaiQik5ZWfB8yhCrdqba_wzooZzmK7EoJ&wd=&eqid=dfb8ae4300001d140000000663314fb4) | College students(16.27 ± 1.02 age)→Young Adults |
| Buke et al., 2021 | 2020.4→During the COVID-19 | Bangladesh→[developing country](https://www.baidu.com/link?url=gpxre6LNEajmcBWVAxBRE_lGvyQEb1T3VICjVuhq7pJ3M_AOsX_3zEhqDwAllNkjmRh5s2EyiUhipyeH7FTCC39izPWoteQDaiQik5ZWfB8yhCrdqba_wzooZzmK7EoJ&wd=&eqid=dfb8ae4300001d140000000663314fb4) | College students(21.36 ± 2.33 age)→Young Adults |
| Abbasi et al., 2021 | 2020.5→During the COVID-19 | China→ [developing country](https://www.baidu.com/link?url=gpxre6LNEajmcBWVAxBRE_lGvyQEb1T3VICjVuhq7pJ3M_AOsX_3zEhqDwAllNkjmRh5s2EyiUhipyeH7FTCC39izPWoteQDaiQik5ZWfB8yhCrdqba_wzooZzmK7EoJ&wd=&eqid=dfb8ae4300001d140000000663314fb4) | College students→Young Adults |
| Islam et al., 2021 | 2020.7→During the COVID-19 | Pakistan→ [developing country](https://www.baidu.com/link?url=gpxre6LNEajmcBWVAxBRE_lGvyQEb1T3VICjVuhq7pJ3M_AOsX_3zEhqDwAllNkjmRh5s2EyiUhipyeH7FTCC39izPWoteQDaiQik5ZWfB8yhCrdqba_wzooZzmK7EoJ&wd=&eqid=dfb8ae4300001d140000000663314fb4) | College students(21.20 ±1.70 age)→Young Adults |
| Ding et al., 2021 | 2020. 9→During the COVID-19 | China→ [developing country](https://www.baidu.com/link?url=gpxre6LNEajmcBWVAxBRE_lGvyQEb1T3VICjVuhq7pJ3M_AOsX_3zEhqDwAllNkjmRh5s2EyiUhipyeH7FTCC39izPWoteQDaiQik5ZWfB8yhCrdqba_wzooZzmK7EoJ&wd=&eqid=dfb8ae4300001d140000000663314fb4) | College students(19.56± 0.95 age)→Young Adults |
| Tanir et al., 2021 | 2020-2021→During the COVID-19 | Taiwan→ [developing country](https://www.baidu.com/link?url=gpxre6LNEajmcBWVAxBRE_lGvyQEb1T3VICjVuhq7pJ3M_AOsX_3zEhqDwAllNkjmRh5s2EyiUhipyeH7FTCC39izPWoteQDaiQik5ZWfB8yhCrdqba_wzooZzmK7EoJ&wd=&eqid=dfb8ae4300001d140000000663314fb4) | College students→Young Adults |
| Guo et al., 2022 | 2020.12-2021. 2→During the COVID-19 | Indian→ [developing country](https://www.baidu.com/link?url=gpxre6LNEajmcBWVAxBRE_lGvyQEb1T3VICjVuhq7pJ3M_AOsX_3zEhqDwAllNkjmRh5s2EyiUhipyeH7FTCC39izPWoteQDaiQik5ZWfB8yhCrdqba_wzooZzmK7EoJ&wd=&eqid=dfb8ae4300001d140000000663314fb4) | College students(19.67±1.62 age)→Young Adults |
| Safffari et al., 2022 | 2021.8-2021.9→During the COVID-19 | Turkey→ [developing country](https://www.baidu.com/link?url=gpxre6LNEajmcBWVAxBRE_lGvyQEb1T3VICjVuhq7pJ3M_AOsX_3zEhqDwAllNkjmRh5s2EyiUhipyeH7FTCC39izPWoteQDaiQik5ZWfB8yhCrdqba_wzooZzmK7EoJ&wd=&eqid=dfb8ae4300001d140000000663314fb4) | College students(22.85 age)→Young Adults |
| Lin et al., 2022 | 2020.8-2021.9→During the COVID-19 | China→ [developing country](https://www.baidu.com/link?url=gpxre6LNEajmcBWVAxBRE_lGvyQEb1T3VICjVuhq7pJ3M_AOsX_3zEhqDwAllNkjmRh5s2EyiUhipyeH7FTCC39izPWoteQDaiQik5ZWfB8yhCrdqba_wzooZzmK7EoJ&wd=&eqid=dfb8ae4300001d140000000663314fb4) | College students(18-22 age )→Young Adults |
| Chen et al., 2022 | 2022.3-2022.4→During the COVID-19 | China→ [developing country](https://www.baidu.com/link?url=gpxre6LNEajmcBWVAxBRE_lGvyQEb1T3VICjVuhq7pJ3M_AOsX_3zEhqDwAllNkjmRh5s2EyiUhipyeH7FTCC39izPWoteQDaiQik5ZWfB8yhCrdqba_wzooZzmK7EoJ&wd=&eqid=dfb8ae4300001d140000000663314fb4) | College students(19.58±1.07  age)→Young Adults |
